# A rare case of multiple spinal epidural abscesses and cauda equina syndrome presenting to the emergency department following acupuncture

**DOI:** 10.1186/s12245-016-0116-5

**Published:** 2016-07-26

**Authors:** Jing Jing Chan, Jen Jen Oh

**Affiliations:** Singapore General Hospital, Outram Road, Singapore, 169608 Singapore

**Keywords:** Acupuncture, *Spinal epidural abscess*, *Cauda equina syndrome*

## Abstract

**Background:**

Acupuncture is a form of traditional Chinese medicine being increasingly used as complementary therapy in many countries. It is relatively safe and rarely associated with deep infections.

**Case presentation:**

In this case report, we describe a middle-aged Chinese patient who presented acutely to our emergency department with cauda equina syndrome secondary to acupuncture-related epidural abscesses, which were treated with surgical decompression and intravenous antibiotics. We also present a review of case reports of this rare condition in available literature.

**Conclusion:**

Emergency physicians should be aware that spinal abscesses may occur after acupuncture, with a broad spectrum of clinical presentations. If a history of recent acupuncture over the symptomatic area is elicited, a high index of suspicion should be maintained and appropriate imaging performed to establish the diagnosis. Treatment is directed by a number of factors, such as severity and duration of neurological deficit and progression of symptoms.

## Background

Acupuncture is a form of traditional medicine that originated in ancient China. It has been reported to be effective in treating various ailments, from depression to hot flashes in cancer [1]. It involves the application of needles into “meridians” to restore the balance of *qi*. However, due to issues with sterility, its use has been associated with the transmission of pathogens, such as hepatitis, human immunodeficiency virus and bacterial skin infections. Rarely, it has also caused pneumothoraces and cardiac tamponade and retained foreign bodies in the abdominal viscera [2].

Herein, we describe a rare case of cervical and lumbar epidural abscesses that developed after needle acupuncture. To our knowledge, only one case of multiple epidural abscesses following acupuncture has been previously reported.

## Case presentation

A 57-year-old woman with no past medical history presented at our emergency department (ED) with 1 day of posterior neck pain, followed by weakness in her left upper limb and both lower limbs, and inability to pass urine since that morning. There was no history of trauma to the head or spine. Two weeks prior to the ED visit, she experienced severe neck ache and consulted a chiropractor, who performed manipulation and needle acupuncture of her back, with relief of her initial symptom.

On examination, the patient was afebrile, with a blood pressure of 116/55 mmHg, a pulse rate of 108 beats per minute, a respiratory rate of 18 breaths per minutes and an oxygen saturation of 97 % in room air. Palpation of her abdomen revealed a bladder distended to the level of the umbilicus. Neurological examination disclosed weakness of the left upper limb and spastic paraparesis. Power in the right upper limb was full. There was also decreased sensation from the level of the neck downwards but with sparing of the right upper limb. A lax anal tone was obtained on digital rectal examination.

X-rays of the cervical and thoracolumbar spine showed disc narrowing at the C5/C6 and C6/C7 levels, and grade 1 spondylolisthesis of L4 on L5. The white blood cell count was 30.13 × 10^9^ cells/L (normal range, 4–10 × 10^9^ cells/L) with neutrophilia, and the C-reactive protein and erythrocyte sedimentation rate levels were 391 mg/L (0.2–9.1 mg/L) and 119 mm/h (3–20 mm/h), respectively. Her procalcitonin level was 2.4 μg/L (<0.5 μg/L).

**Fig. 1:**
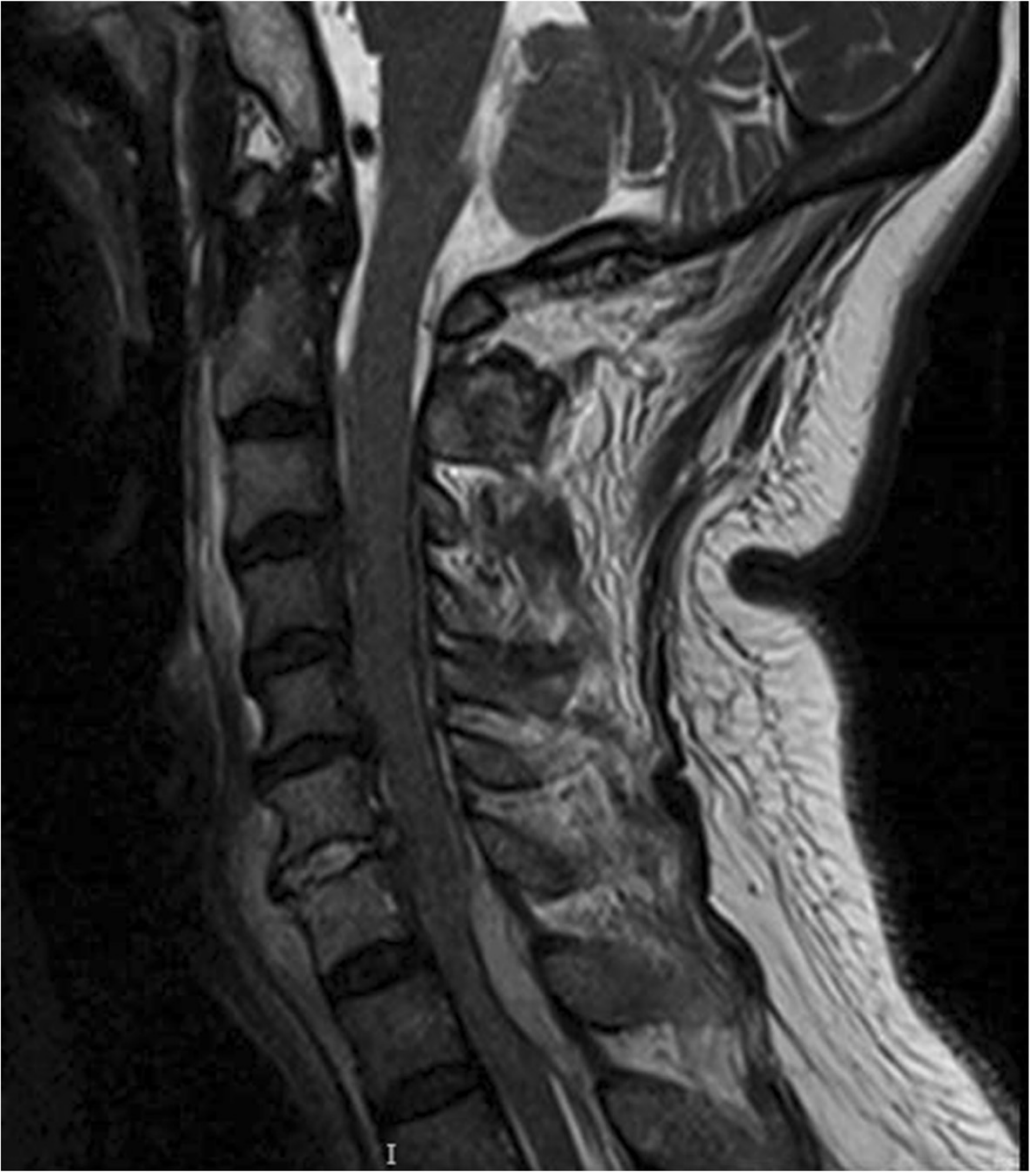
Sagittal view of the cervical spine on MRI, showing the epidural abscess which is compressing on the cervical cord anteriorly

**Fig. 2:**
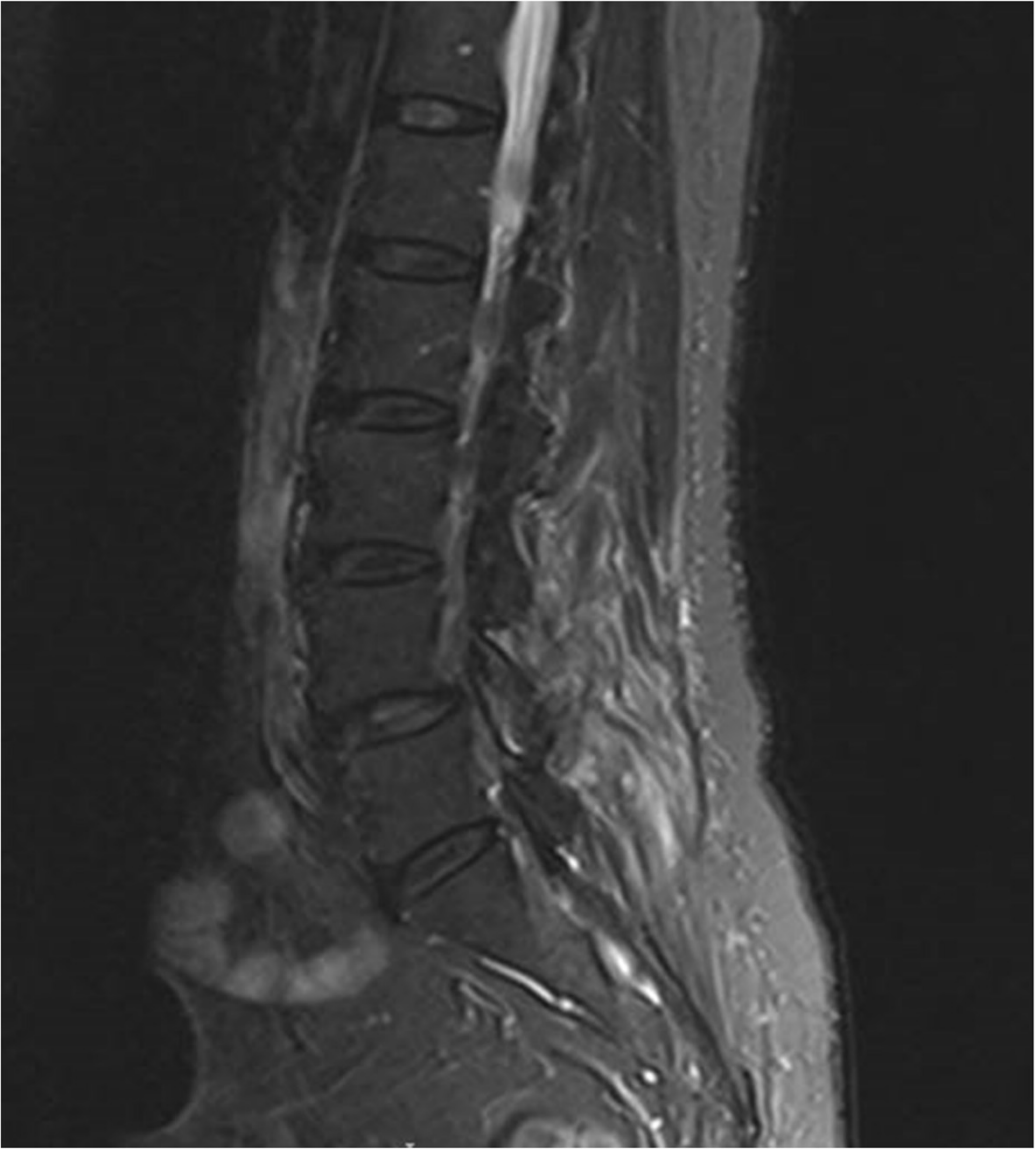
There was high T2 signal and enhancement seen in the left inferior facet of L4 suspicious for osteomyelitis

Magnetic resonance imaging (MRI) of the spine showed multiple spinal abscesses in the posterior epidural space from C5 to T1 and from L4 to L5. The former measured 0.7 × 1.3 × 5.1 cm, displacing the cervical cord anteriorly and compressing it at C7-T1, with a high T2 signal intensity in the cord (see Fig. 1). There was also a high T2-TIRM signal in the C6-7 vertebral bodies and intervening disc with abnormal enhancement, in keeping with osteomyelitis and discitis. This was also seen in the lumbar spine (see Fig. 2).

**Fig. 3:**
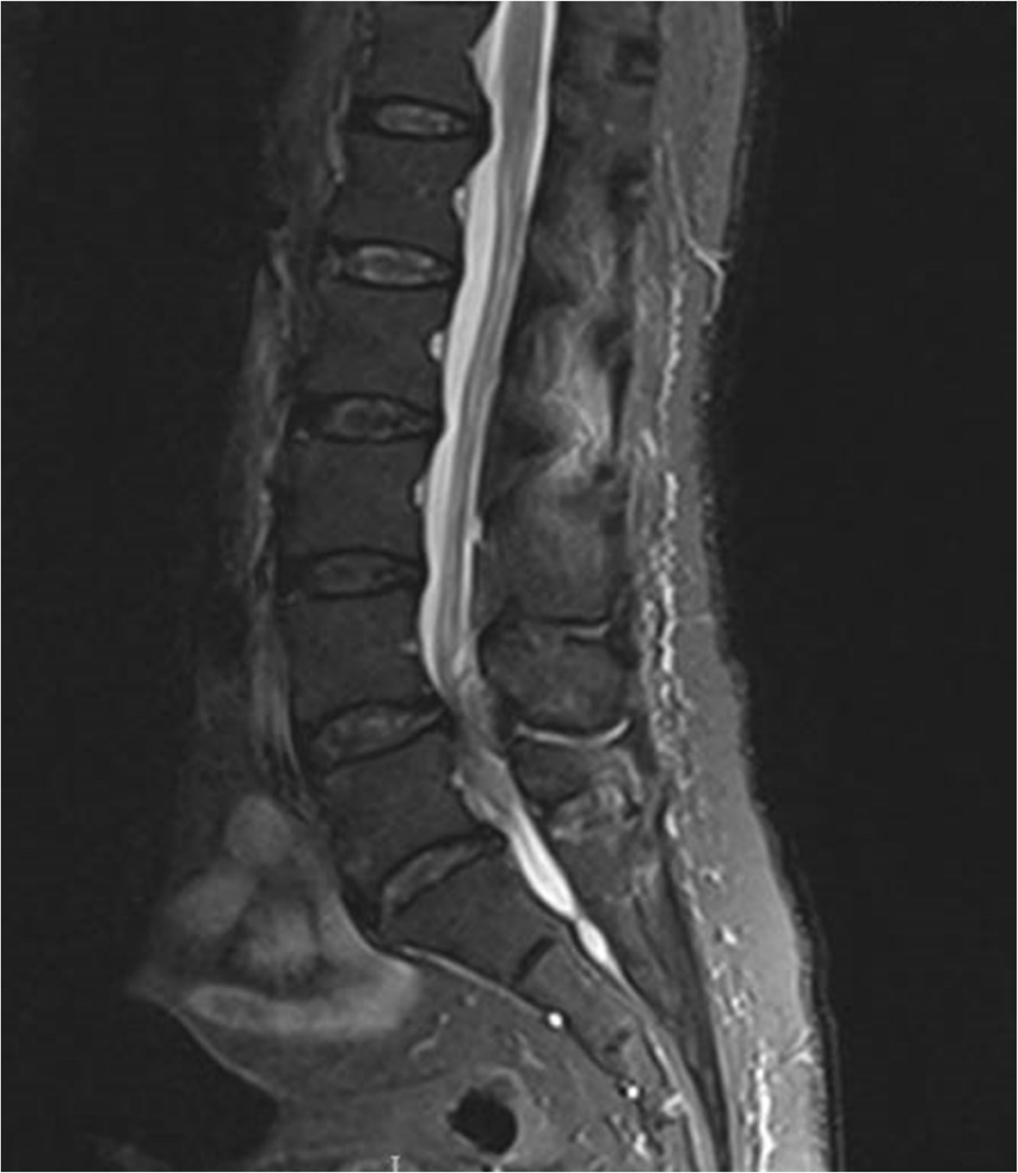
The collection in the posterior epidural space and the posterior disc bulge is seen compressing the thecal sac and the cauda equina

In the lumbar spine, another collection measuring 0.4 × 1.2 × 3 cm was noted in the right posterior epidural space along the right lamina of L4/L5. Together with a disc bulge, this compressed the thecal sac and cauda equina (see Fig. 3).

She was admitted to the orthopaedic surgery high dependency unit, and intravenous (IV) antibiotics were commenced. Surgical decompression of the spinal cord and drainage of the epidural abscesses was performed early the next day. Blood cultures and intra-operative fluid cultures grew pan-sensitive *Staphylococcus aureus*. Following advice from the infectious disease specialist, the patient completed 6 weeks of IV penicillin G. Repeat blood cultures taken 1 week after initiation of antibiotics were negative.

Post-surgery, she underwent intensive physical therapy for a month, after which she was discharged independent without aid in the community. A follow-up MRI scan performed 4 months later showed resolution of all the abscesses.

### Discussion

A spinal epidural abscess (SEA) is a pyogenic infection of the potential space between the vertebral body and dura mater of the spine. Although rare, their incidence has been rising gradually over the past decade, possibly due to increasing intravenous drug abuse, an ageing population with multiple comorbidities and more frequent use of spinal instrumentation.

SEAs can be either primary or secondary. Primary SEAs result from haematogenous spread of pathogens from a distant focus to the epidural space, while secondary SEAs occur after spinal trauma, injections, surgery or direct inoculation of a pathogen into the epidural space [3].

In a literature review of 40 studies published from years 2000 to 2013 [4], the most common symptoms and signs reported in SEA were neck and back pain, fever and neurological deficits. Altered mental state and incontinence were also documented. However, presentation can be ambiguous and variable, resulting in delayed diagnosis. The most common predisposing factor is diabetes mellitus. Others include alcoholism, renal failure, intravenous drug abuse, chronic inflammatory conditions, bacteremia and previous spinal intervention.

The neuroimaging study of choice is MRI. It visualizes soft tissues as effectively as computed tomography (CT) myelography, with a comparable sensitivity for SEA. It also provides superior identification of perimedullary lesions with no risk of introducing pathogens into the thecal sac. MRI is able to differentiate between SEAs and other spinal lesions such as subdural abscesses and tumours. The classical description of an SEA on MRI is the collection in the epidural space which is iso- or hyperintense on T1 images which enhances with gadolinium contrast, and a T2 image with a non-homogeneous and hyperintense signal [4].

A systematic review of 12 studies published after 1999 [5] reported *S. aureus* as the most common causative pathogen, followed by *Streptococcus* species.

SEA formation following acupuncture is rare, and a literature search yielded only seven such cases between 1998 and 2015, summarized in Table 1. There is variability in patient profile, clinical presentation, spinal level of involvement, causative organism and treatment.

**Table 1:** Case reports of spinal epidural abscess formation following acupuncture published between 1998 and 2015

Reference	Age, gender	Spine level	Signs and symptoms	Pathogen	Treatment
9	67, M	C1-2	Fever, posterior nuchal and back pain	No specific pathogen	Antibiotics
10	64, M	T11-L3	Severe back pain	*Escherichia coli*	Antibiotics
11	13, M	L4-5	Severe back pain, fever	No specific pathogen	Antibiotics
12	47, M	C1-3	Posterior nuchal pain and swelling	No specific pathogen	Antibiotics
13	19, M	C2-6	Progressive neck stiffness and fever	Group B *Streptococcus*	Antibiotics
14	80, F	C3-7, L3-5, L5-S1	Fever, progressive quadriparesis, difficulty voiding	*Staphylococcus aureus*	Surgical drainage + antibiotics
15	47, M	L3-5	Fever, low back pain, right sciatica	*Serratia marcescens*	Surgical decompression + antibiotics
Current case	57, F	C5-T1, L4-5	Posterior nuchal pain, inability to void, weakness in both lower limbs and left upper limb	*Staphylococcus aureus*	Surgical decompression + antibiotics

Acupuncture is an early form of medical intervention that originated in China thousands of years ago and continues to be practised today, especially in Asian countries. Studies report its clinical benefits for chronic painful musculoskeletal disorders, headaches and hypertension. Major complications are uncommon, with an incidence of 0.55 per 10,000 individual patients in 12 prospective surveys [6]. The most common infection is hepatitis B (>60 %) due to transmission via contaminated needles. Only three cases of spinal infection were reported in this review.

Treatment of SEA includes antibiotics for systemic manifestations and surgery for local disease control. One literature review of 28 case series acknowledges that SEAs are rare, with significant variation in their causes, anatomical locations and rate of progression, making it difficult to build a strong evidence base to stratify patients to the most effective treatment algorithm [7].

However, there is relative consensus that patients with acute or progressive neurological deficit, progressive deformity, spinal instability, or disease progression despite antibiotics require surgical intervention [7].

When surgery is required, a posterior laminectomy is the most common approach. Less invasive methods of surgical evacuation include CT-guided needle aspiration, hemilaminectomy and interlaminar fenestration, which report promising results. For extensive SEA, suction irrigation catheters passed through end- or intermediate-level laminectomies or multilevel unilateral fenestrations may be performed to avoid a multilevel laminectomy [7].

Based on the best available evidence, medical management should be considered in the following patients [7]:

- Those who are unfit for surgery (medically unstable, serious comorbidities)
- Complete paralysis for >48 h without significant concern for an ascending lesion (surgical risks likely to outweigh the chance of functional recovery)
- Risk factors for failure of medical treatment
  - Diabetes mellitis
  - Bacteremia
  - White blood cell count >12.5 × 10^9^ cells/L
  - C-reactive protein >115 mg/L
  - Ring enhancement of lesion on MRI
  - Methicillin-resistant *S. aureus* infection

Although the period of antibiotic therapy is debatable, this should last at least 4 weeks because studies have shown a 25 % rate of relapse in patients who were treated for less than 4 weeks [4].

The patient and treating physician should bear in mind that even in the absence of risk factors, there is no guarantee that medical treatment will not fail and that the patient will not require surgery eventually. The risk of failed medical management has been reported to be between 8.3 and 17 %. The best predictor of post-operative neurological outcome is preoperative neurological status [7].

## Conclusion

Although acupuncture is commonly practised in Singapore, there are no strict guidelines regarding skin decontamination before needle insertion, and there is no specific research on this topic. Most adverse effects of acupuncture appear to be secondary to low hygiene standards, insufficient basic medical knowledge and inadequate acupuncture training [8]. Therefore, acupuncturists should be educated about the importance of infection control measures. Ideally, the needling sites should be swabbed with 70 % ethyl or isopropyl alcohol, and the solution should be allowed to dry prior to needle insertion. The practitioner should also ensure a clean working environment, the use of sterile needles, thorough hand washing before and after treating patients and careful management of used needles.
